# Exploration of *Aspergillus fumigatus* Ras pathways for novel antifungal drug targets

**DOI:** 10.3389/fmicb.2015.00128

**Published:** 2015-02-26

**Authors:** Qusai Al Abdallah, Jarrod R. Fortwendel

**Affiliations:** Department of Microbiology and Immunology, University of South Alabama, Mobile, AL, USA

**Keywords:** Ras protein, G domain, hypervariable region, post-translational modifications, spatio-temporal regulation, fungal pathogenesis, antifungal therapeutics

## Abstract

Ras pathway signaling is a critical virulence determinant for pathogenic fungi. Localization of Ras to the plasma membrane (PM) is required for Ras network interactions supporting fungal growth and virulence. For example, loss of *Aspergillus fumigatus* RasA signaling at the PM via inhibition of palmitoylation leads to decreased growth, altered hyphal morphogenesis, decreased cell wall integrity and loss of virulence. In order to be properly localized and activated, Ras proteins must transit a series of post-translational modification (PTM) steps. These steps include farnesylation, proteolytic cleavage of terminal amino acids, carboxymethylation, and palmitoylation. Because Ras activation drives tumor development, Ras pathways have been extensively studied in mammalian cells as a potential target for anti-cancer therapy. Inhibitors of mammalian Ras interactions and PTM components have been, or are actively being, developed. This review will focus on the potential for building upon existing scaffolds to exploit fungal Ras proteins for therapy, synthesizing data from studies employing both mammalian and fungal systems.

## INTRODUCTION

*Aspergillus fumigatus* is the most common fungal pathogen of invasive aspergillosis in immunocompromised patients. Despite the introduction of several antifungal drugs, infections related to invasive aspergillosis are usually severe and fatal (Latge, 1999). Therefore, there is an increasing demand for new drugs against *A. fumigatus* infections, and thereby identification of target proteins for therapeutic drug design.

Ras-mediated signaling pathways play key roles in regulating cell responses to different stresses via a wide range of effector proteins (for more information, refer to Rajalingam et al., 2007). In pathogenic fungi, Ras signaling pathways control virulence in host cells (reviewed in detail in Fortwendel, 2012). Therefore Ras proteins, and their effectors, represent potential targets of intervention for novel antifungal therapies. Due to their role in tumor formation, mammalian Ras post-translational modification (PTM) pathway proteins have been studied in detail for their potential as targets for anticancer therapeutics (refer to Downward, 2003; Adjei and Hidalgo, 2005; Spiegel et al., 2014). In this minireview, we discuss these studies in the context of development of antifungal therapy. Since RasA is the major Ras protein in *A. fumigatus*, this brief review focuses on the RasA signaling pathway.

## *A. Fumigatus* Ras PROTEINS

Ras proteins are low molecular weight monomeric G-proteins, which localize to the plasma membrane (PM) and exhibit GTPase activities (Wennerberg et al., 2005). They are induced by extracellular stimuli and function primarily as signal mediators for several downstream cascades. Such cascades activate transcription factors, which control a wide range of cellular processes such as cell growth, division, differentiation and survival (Weeks and Spiegelman, 2003). In contrast to human cells, which typically express three Ras isoforms (HRas, KRas, and NRas), only two Ras homologs (RasA and RasB) are produced in *A. fumigatus* (Fortwendel et al., 2004). Based on sequence similarity, RasA is more closely related to the human H-Ras with homologs found in most eukaryotes. In contrast, RasB is only produced by filamentous fungi (Fortwendel et al., 2004). Both, RasA, and RasB, exhibit distinct but overlapping roles in conidial germination, mycelial growth, conidiogenesis, and cell mitosis (Fortwendel et al., 2004, 2005, 2008, 2012). Additionally, both proteins modulate virulence in *A. fumigatus* and other pathogenic fungi (Fortwendel et al., 2005, 2012; Fortwendel, 2012).

## DOMAIN STRUCTURE OF Ras PROTEINS

The domain structure of human Ras proteins has been reviewed in detail previously (Sprang, 1997; Vetter and Wittinghofer, 2001; Wittinghofer and Vetter, 2011). Briefly, the approximately 190-amino acid protein is divided into 165 highly conserved amino acids (90–100% identical) at the N-terminus (known as the G domain or GTPase domain) and a C-terminal hypervariable region (HVR) that encompasses the remaining amino acids (Hancock, 2003). In the next sections, we will discuss the domain structure of both regions and their role in mediating Ras activation, transmembrane localization and cell signaling.

### THE G DOMAIN FACILITATES PROTEIN CONFIRMATION AND DOWNSTREAM SIGNALING

Numerous biochemical, molecular and structural studies, involving both yeast and mammalian cells, have shown that the G domain of Ras-like proteins houses the amino acid sequences required for binding guanine nucleotides [i.e., guanosine diphosphate (GDP) and guanosine triphosphate (GTP)], GTPase-activating protein (GAP), guanine nucleotide exchange factor (GEF), and downstream effectors (Ahearn et al., 2012). The G domain is organized into six β sheets and five α helices. Additionally, two loop regions, designated switch I and switch II, mediate Ras transformation between its two interchangeable activity states via conformational change during binding of guanine nucleotides (Vetter and Wittinghofer, 2001).

The Ras activation mechanism involves GEF proteins, which promote the release of GDP. GTP, which exists in the cytoplasm at concentrations 10 times higher than that of GDP, binds to the GDP-free form of Ras. GTP association with Ras releases energy, which changes protein conformation at the switch I and II regions. This transforms Ras to the active state and allows binding of the effector proteins to its G domain. Active Ras proteins are negatively regulated by GAP proteins. Binding of GAP to Ras protein increases its intrinsic GTPase activity 10^5^ fold and hydrolyzes GTP to GDP. The hydrolysis of GTP depletes the released energy, causing conformational changes at the switch domains, and subsequently releasing the effector (Vetter and Wittinghofer, 2001; Wennerberg et al., 2005; Kyriakis, 2009; Ahearn et al., 2012; Prior and Hancock, 2012).

### THE HVR GOVERNS Ras MEMBRANE LOCALIZATION AND ANCHORING

The HVR of RasA homologs is divided into two regions: an anchor and a linker. The anchor region is highly conserved among Ras isoforms and is composed of a CAAX box—where C is cysteine, AA are two aliphatic amino acids, and X is any amino acid—and a palmitoylation motif (Gao et al., 2009).

**FIGURE 1:**
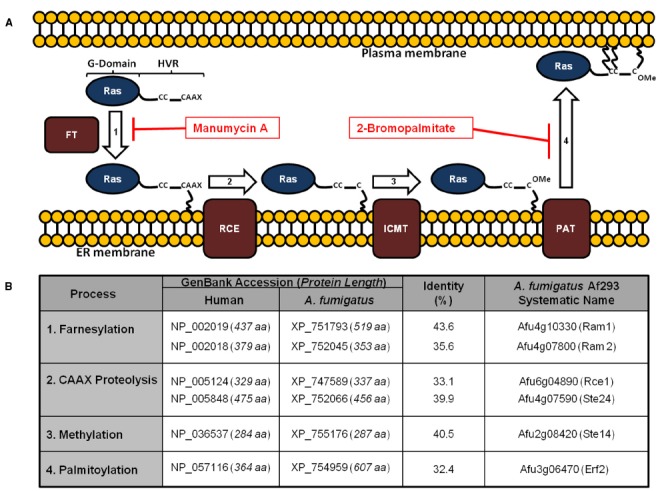
**Conservation of the Ras post-translational modification pathway in *Aspergillus fumigatus*. (A)** Ras proteins transit a series of post-translational modifications to reach the plasma membrane. These include: (1) farnesylation of cytoplasmic Ras on a conserved cysteine residue by a dual subunit, protein farnesyltransferase enzyme complex; (2) cleavage of the C-terminal CAAX motif; (3) methylation of the processed C-terminus; and (4) palmitoylation of conserved cysteine residues upstream of the CAAX motif. Farnesylation is prerequisite for association with the endoplasmic reticulum, whereas palmitoylation is required for stable association with the plasma membrane. Inhibitors with activity against these processes in *A. fumigatus* include manumycin A and 2-bromopalmitate, targeting farnesylation and palmitoylation, respectively. FT = farnesyltransferase; RCE = Ras converting enzyme; ICMT = isoprenylcysteine carboxymethyltransferase; PAT = palmitoyltransferase. **(B)** Homologs of the protein components of the Ras post-translational modification pathway are shown. Protein lengths in amino acids (aa) are included with the GenBank accession numbers. Identity (%) was determined using protein alignments in Lasergene software (DNAstar). For reference, homologs of the yeast pathway are given in parentheses next to the *A. fumigatus* Af293 systematic name (right column).

The anchor plays an essential role in Ras subcellular trafficking and membrane localization. Protein trafficking and subsequent membrane association of many proteins is typically mediated by hydrophobic transmembrane domains. However, Ras proteins lack such domains, and therefore the protein undergoes several PTMs at the CAAX box and the palmitoylated cysteine motif which convert the HVR to a hydrophobic, membrane-associated domain (Figure 1A) (Takai et al., 2001; Hancock, 2003; Larsen et al., 2006; Iwasaki and Ōmura, 2007).

Ras PTM mechanisms have been studied in detail in human and yeast cells. In spite of lack of similar studies in *A. fumigatus*, homologous proteins have been identified in the *A. fumigatus* genome (Figure 1B), implying conservation of RasA PTM processes in *Aspergillus* species. The first step of the Ras PTM series is prenylation, which is the process of covalent addition of a farnesyl (farnesylation) or geranylgeranyl group (geranylgeranylation) at the cysteine residue of the CAAX box via farnesyl transferase (FT) or geranylgeranyl transferases (GGT I and II), respectively (Berndt and Sebti, 2011). Prenylation of the CAAX box facilitates the association of Ras protein to the endoplasmic reticulum (ER) membrane (Omerovic et al., 2007). At the ER membrane, the farnesylated (or geranylgeranylated) Ras protein is further processed by AAX cleavage via type I (Ste24) and type II CAAX prenyl endopeptidase (Rce1) (Manolaridis et al., 2013).

The remaining prenylated cysteine residue of the CAAX box is then methylated by isoprenylcysteine carboxyl methyltransferase (ICMT) in the ER (Chiu et al., 2004). Prenylation, proteolysis, and methylation suffice weak binding of the CAAX motif cysteine residue to the ER membrane. Such unstable association causes Ras to encounter a constant exchange between the ER membrane and the cytoplasm (Greaves and Chamberlain, 2007). Therefore, a second moiety is required to stabilize Ras association to the ER membrane. Such a signal varies and can be a lysine polybasic domain in K-Ras(B), a single palmitoylation site in N-Ras and K-Ras(A), or a double palmitoylation site in H-Ras (Hancock et al., 1989, 1990). Palmitoylation, specifically S-palmitoylation, is the addition of palmitate to the cysteine residue(s) via thioester bond (Smotrys and Linder, 2004; Wan et al., 2007). In Ras proteins, palmitoylation cysteines are located in the anchor region and are adjacent to the CAAX box cysteine residue (Linder and Deschenes, 2007). Palmitoylated Ras is transported from the Golgi to the PM via the exocytic vesicular pathway (Goodwin et al., 2005). Unlike prenylation, palmitoylation is a reversible step, as Ras can be depalmitoylated via thioesterase on the PM. Depalmitoylated Ras recycles back to the Golgi via a non-vesicular pathway. This cycle of palmitoylation and depalmitoylation is used by the cell to avoid unnecessary accumulation of Ras proteins on the PM (Goodwin et al., 2005; Salaun et al., 2010).

In fungi, the amino acid composition of the anchor region varies among species in different ways. First, the number of cysteine residues in the palmitoylation region varies from one cysteine in *S. cerevisiae*, *S. pombe*, and *Candida albicans*; to two cysteine residues in *Cryptococcus neoformans* and other filamentous fungi such as *A. fumigatus*. Second, variations in the anchor region might exist between different Ras homologs within one fungal species. For example, unlike RasA, *A. fumigatus* RasB does not contain the palmitoylation cysteine residues. Although no experimental evidence for RasB localization exists, the differences in anchor region amino acid sequence might indicate different subcellular localizations of RasA and RasB (Fortwendel, 2012).

## Ras-MEDIATED FUNGAL MORPHOGENESIS AND VIRULENCE DEPEND ON THE SPATIO-TEMPORAL REGULATION OF Ras ACTIVITY

Like their human counterparts, the cellular functions of fungal Ras GTPases depend on the spatio-temporal regulation of the protein on the PM. Understanding the spatio-temporal organization of RasA has been a necessary step in selecting potential targets that are predicted to interfere with its ability to properly transmit signals for growth, stress response and virulence. The following sections will briefly summarize our current knowledge of RasA spatial and temporal regulation mechanisms.

### THE TEMPORAL REGULATION OF RasA ACTIVITY

The essential role of temporal activation of RasA has been investigated by comparing the phenotype of *A. fumigatus* mutants that express dominant negative RasA (DNrasA) or dominant activate RasA (DArasA) to that of a *rasA* (Δ*rasA*) deletion mutant. The DNrasA and DArasA mutants provide the ability to study the phenotypic effects of improper temporal inactivation or activation of RasA, respectively, during development. DNrasA, and Δ*rasA* strains show similar phenotypes (i.e., delayed in germination), whereas the DArasA mutant initiates germination in the absence of a germinant. Additionally, mycelia of all three strains grow slower than wild type and exhibit defects in polarity maintenance. Although constitutive Ras activation delays germ tube formation and reduces colony outgrowth, the DArasA mutant also displays hyphal swelling and spontaneous lysis during fully polarized growth (Fortwendel, 2012). These data show the importance of temporal regulation of RasA activity for proper hyphal morphogenesis, since the inability to modulate RasA activity during developmental progression causes abnormalities in fungal growth.

### THE SPATIAL REGULATION OF RasA SIGNALING

In addition to the previously described temporal regulation, spatial regulation of Ras signaling plays an essential role in RasA function. Evidence for the cellular mechanisms that control *A. fumigatus* RasA PTM and localization was obtained from subcellular localization analyses of RasA using GFP tagging. Similar to human Ras, RasA localizes to the PM of *A. fumigatus*. However, when RasA farnesylation is blocked by exchange mutagenesis of the CAAX box cysteine residue (C210) to serine, RasA aborts the PM localization and accumulates in the cytoplasm. Additionally, expression of RasA in an *A. fumigatus* deletion strain of the putative palmitoyltransferase subunit gene (Δ*erfD*) shows a punctate localization of RasA, implying palmitoylation of RasA is required for PM localization. Furthermore, mutation of the palmitoylation double cysteine motif (C206 and C207) to serine mislocalizes RasA to endomembranes. Phenotype analysis of these mutants showed that *A. fumigatus* strains expressing either a non-farnesylated or non-palmitoylated RasA exhibit a full or partial Δ*rasA* phenotype, respectively (Fortwendel et al., 2012; Norton and Fortwendel, 2014). Consistent with these data, similar results have been obtained in other pathogenic fungi. For example, in *C. neoformans*, farnesylation and palmitoylation are both required for normal Ras1 localization and morphogenesis (Nichols et al., 2009). In *C. albicans*, both PTMs are required for localization, but farnesylation plays the greater role in Ras-mediated growth and morphogenesis (Piispanen et al., 2011). Taken together, these data support the importance of RasA PTM processes for fungal growth and virulence.

## APPROACHES FOR DEVELOPING Ras-TARGETED ANTIFUNGAL THERAPEUTICS

Mammalian Ras signaling pathways have been the target of extensive research for developing anticancer therapeutics. This accumulated knowledge could be translated into novel strategies to treat *Aspergillus* infections since the mechanisms of Ras activation and PTMs are shared by both human and fungi. The development of Ras-targeted anticancer therapy has focused on: (1) targeting Ras proteins directly, (2) blockade of Ras upstream and downstream signaling pathways, and (3) inhibition of Ras PTMs. There are many Ras inhibitors that have tested in mammalian cell as anticancer therapeutics. Details on specific successes and failures have been reviewed extensively (Adjei and Hidalgo, 2005; Berndt et al., 2011; Spiegel et al., 2014). Although all three of these have been pursued as potentially viable channels for inhibition of Ras function, most progress has been achieved in the development of anti-Ras compounds targeting the PTM pathway. In the following section, we will briefly describe these approaches, focusing mainly on inhibition of Ras PTMs and the published data that support this pathway as a potential anti-*Aspergillus* therapeutic target.

### TARGETING Ras PROTEINS AND Ras PROTEIN INTERACTIONS

In *A. fumigatus*, RasA modulates fungal pathogenesis and has been considered an attractive target for antifungal agents. Directly targeting Ras proteins for anticancer therapies has proven a difficult task. For example, inhibition of Ras-GTP binding is difficult to achieve, since the Ras-GTP interaction is very high affinity, occurring in the picomolar range (Gysin et al., 2011). However, recent approaches have generated small molecules that inhibit K-Ras activation (Maurer et al., 2012). This method relied on in-depth structural analyses of the K-Ras protein, the level of which have not been accomplished for any fungal Ras protein. As such, the applicability of such inhibitors remains unknown for fungal Ras proteins.

In general, fungal Ras proteins appear to share similar signaling mechanisms with their human homologs, and therefore inhibitors of these signaling events might also have great potential for thwarting invasive fungal infections. Examples of these are protein kinases that modulate downstream Ras signaling pathways. Several kinase inhibitors have been applied successfully to treat cancer (reviewed in detail by Downward, 2003), however, these molecules have not been tested in models of fungal infection. Although the signaling mechanisms are similar between humans and fungi, a deeper understanding of the biochemistry underpinning Ras-mediated signaling in pathogenic fungi is required for the identification of fungal-specific, selective targets in this area.

### INHIBITION OF Ras PTM

#### Inhibition of farnesylation

In contrast to the limited number of therapeutics directly targeting Ras proteins, multiple compounds are being developed and employed to inhibit steps in the PTM pathway of human Ras proteins. Importantly, the individual elements of the PTM pathway are conserved between humans and fungal pathogens, the first step of which is the lipidation of Ras with a farnesyl moiety (Figure 1B). There are several farnesyl transferase inhibitors (FTIs) that are used or being clinically tested for use as anticancer agents (described in detail by Appels et al., 2005). The antifungal activities of some of these FTIs have been evaluated against several pathogenic fungi. For example, when the wild type strain of *C. neoformans* was treated with six different FTIs, one inhibitor, i.e., Manumycin A, showed inhibitory activities comparable to Amphotericin B—an antifungal agent. Additionally, when a *C. neoformans* mutant that lacks the cell wall capsule, i.e., cap59 mutant, was used, two additional FTIs, namely ethylenediamine inhibitor #2 and tipifarnib, showed elevated inhibitory activities. Interestingly, treatment of *C. neoformans* with high concentrations of Manumycin A caused a shift in Ras1 localization from the PM to the cytosol (Hast et al., 2011). Similar experiments have been carried out to study antifungal activities of Manumycin A against several species from *Aspergillus* and *Candida*. Compared to *C. neoformans*, the FTI minimal inhibitory concentrations (MIC) were 80–160-fold and 5–10-fold higher for *Aspergillus* and for *Candida*, respectively. (Hast et al., 2011; Qiao et al., 2013). However, it is unclear whether such differences in MIC are caused by fungal resistance to Manumycin A; or variations in experimental procedure, media composition, or pH of the media. In a similar experiment, the inhibition of protein farnesylation in *C. albinans* by farnesyl transferase inhibitor III (FPT inhibitor III) blocked the development of yeast to hyphae (McGeady et al., 2002), which is a Ras-mediated virulence step (Cutler, 1991; Feng et al., 1999). Additionally, FPT Inhibitor III blocks hyphal differentiation in a dose-dependent manner in *C. neoformans* (Vallim et al., 2004).

To better understand the protein–protein interactions between FT and their inhibitors (FTIs), structural studies of inhibitor-bound *A. fumigatus* and *C. neoformans* FT were compared to their human homolog. These studies revealed that the substrate-binding groove residues are highly conserved between human and fungal FT, while the product exit groove displays a high sequence divergence. Importantly, these studies reveal fungal-specific attributes of the highly conserved Ras PTM pathway. For example, both grooves are wider in fungal FT in comparison to their human homolog, causing weaker binding activities of inhibitors toward *Aspergillus* FT in comparison to human FT (Hast et al., 2011; Mabanglo et al., 2014). Therefore, modification of anticancer FTIs is required for optimal antifungal efficiency. Re-purposing FTIs developed for anticancer treatments may represent a novel area for antifungal drug development.

In addition to targeting Ras farnesylation, mapping the fungal farnesylome, i.e., proteins that are farnesylated by farnesyltransferase, will open the door for numerous potential drug targets. One example is the Ras-related protein, Rheb, which is, like Ras proteins, farnesylated before transmembrane localization (Clark et al., 1997). The cellular functions of mammalian Rheb are inhibited by cell treatment with FTI (Castro et al., 2003). In *A. fumigatus*, the Rheb homolog, i.e., RhbA, plays an important role in fungal pathogenesis and vegetative growth. An *A. fumigatus rhbA* deletion mutant, Δ*rhbA*, exhibits impaired virulence in mouse model and reduced growth on minimal media supplemented with poor nitrogen sources. Additionally, this strain displays higher sensitivity to the rapamycin antibiotic, which inhibits TOR kinases (Panepinto et al., 2003). Taken together, this suggests that fungal Rheb, i.e., Rhb, could serve as potential target for antifungal therapy.

#### Inhibition of palmitoylation

Palmitoylation is another putative target for antifungal therapeutics since RasA palmitoylation is important for mycelial polarized growth and virulence. Targeting Ras palmitoylation is still at the beginning stages as an anticancer therapeutic. Palmitoylation inhibitors have only recently been developed and have not yet been employed in disease models (reviewed in Chavda et al., 2014). To our knowledge, only one study assessed the potential of inhibition of RasA palmitoylation as antifungal target. In this study, blocking RasA palmitoylation in *A. fumigatus* by 2-bromopalmitate disrupts RasA transmembrane localization and reduces fungal growth in liquid culture (Fortwendel et al., 2012). Therefore, additional studies that aim at designing, developing and assessing novel compounds that target fungal RasA palmitoylation are warranted.

## CONCLUSION

The potential for developing antifungal therapeutics by targeting the Ras signaling pathway is a promising avenue of research. Ras signaling has been studied intensively in humans, and the accumulated knowledge can be utilized as a scaffold for the development of antifungal agents with selective toxicity. In support of this, the individual components of the Ras PTM pathway share only partial sequence similarity with their human homologs (Figure 1B). Additionally, further characterization of Ras regulatory pathways in pathogenic fungi is necessary to deepen our understanding of fungal growth and virulence.

### Conflict of Interest Statement

The authors declare that the research was conducted in the absence of any commercial or financial relationships that could be construed as a potential conflict of interest.
